# The gill epithelial cell lines RTgill-W1, from Rainbow trout and ASG-10, from Atlantic salmon, exert different toxicity profiles towards rotenone

**DOI:** 10.1007/s10616-022-00560-0

**Published:** 2022-11-17

**Authors:** Anita Solhaug, Mona Gjessing, Morten Sandvik, Gunnar Sundstøl Eriksen

**Affiliations:** 1 Chemistry and Toxinology, Norwegian Veterinary Institute, P.O Box 64, 1431 Ås, Norway; 2 Aquatic Biosecurity, Norwegian Veterinary Institute, P.O Box 64, 1431 Ås, Norway

**Keywords:** Rotenone, RTgill-W1, ASG-10, ROS, Glutathione

## Abstract

In order to ensure the proper use and interpretation of results from laboratory test systems, it is important to know the characteristics of your test system. Here we compare mitochondria and the handling of reactive oxygen species (ROS) in two gill epithelial cell lines, the well-known RTgill-W1 cell line from Rainbow trout and the newly established ASG-10 cell line from Atlantic salmon. Rotenone was used to trigger ROS production. Rotenone reduced metabolic activity and induced cell death in both cell lines, with RTgill-W1 far more sensitive than ASG-10. In untreated cells, the mitochondria appear to be more fragmented in RTgill-W1 cells compared to ASG-10 cells. Furthermore, rotenone induced mitochondrial fragmentation, reduced mitochondria membrane potential (Δψm) and increased ROS generation in both cell lines. Glutathione (GSH) and catalase is important to maintain the cellular oxidative balance by eliminating hydrogen peroxide (H_2_O_2_). In response to rotenone, both GSH and catalase depletion were observed in the RTgill-W1 cells. In contrast, no changes were found in the GSH levels in ASG-10, while the catalase activity was increased. In summary, the two salmonid gill cell lines have different tolerance towards ROS, probably caused by differences in mitochondrial status as well as in GSH and catalase activities. This should be taken into consideration with the selection of experimental model and interpretation of results.

**Graphical abstract:**

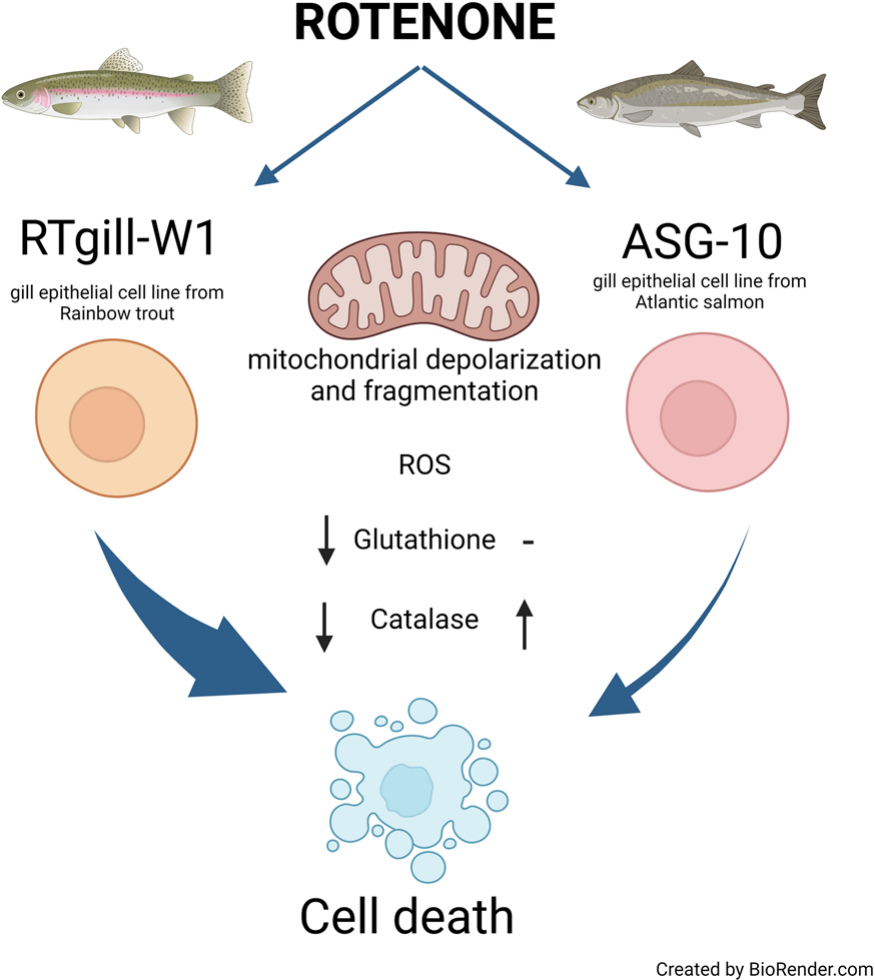

**Supplementary Information:**

The online version contains supplementary material available at 10.1007/s10616-022-00560-0.

## Introduction

While respiration is the gills primary function, the gill is a physiologically diversified organ and compromised gills may have a broad impact on the animal. According to the fish health report, 50 million (15.2%) farmed Norwegian Atlantic salmon (*Salmo salar* L.) died after sea transfer in 2021 (Sommerset et al. 2021). The causations of these deaths are diverse, with gill diseases being part of the explanation. Complex gill disease or complex gill disorder (CGD) are terms used to describe gill disease manifestations in which the histopathological pattern is complex and partly overlaps with multifactorial gill disease where different gill diseases are concurrently present. The list of agents infecting gills of salmon is long and the pathogenicity of the individual agents is poorly understood. Current knowledge demonstrate that suffering and losses related to gill diseases must be solved and good models are needed. Furthermore, there is currently a drive in the society towards reducing the number of experimental animals including fish (Paparella et al. 2021). Therefore, it is desirable to develop good and relevant in vitro models to study gill health and the impact of chemical and biological factors on gill functions. It is important to know the potentials and limitations in such a model system. Comparative knowledge reduces uncertainties when findings are extrapolated between species in a risk assessment (Bennekou 2019). Oxidative stress is frequently involved in both toxicological insult and infections. Consequently, knowledge of a model systems ability to handle oxidative stress is highly relevant for the interpretation of results using in vitro model systems.

Rotenone is well known for its ability to kill fish and is frequently used to eradicate undesired fish populations. Also, plant materials containing rotenone has traditionally been used for fishing by indigenous people (Said et al. 2020). Rotenone is highly toxic to fish and certain invertebrates and has in the recent years been used to exterminate invasive fish species and parasites in Norwegian rivers (Sandodden et al. 2018, 2022).

**Fig. 1:**
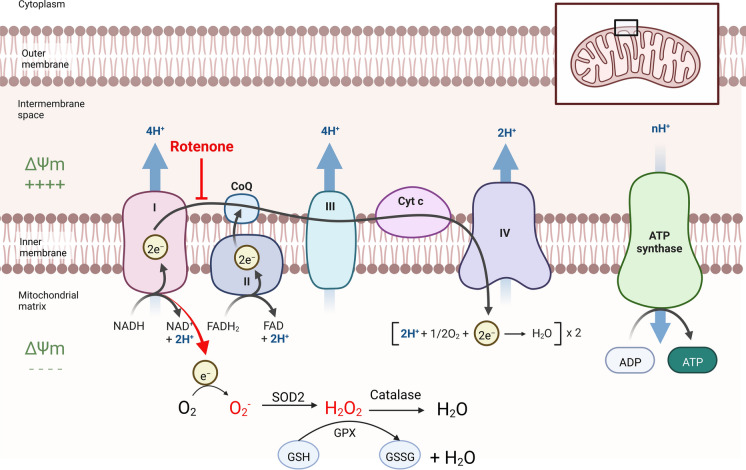
Mitochondrial transport chain. Rotenone inhibits complex 1 in the mitochondrial transport chain, which leads to the production of ROS, reduced mitochondrial membrane potential (ΔΨm) and reduced ATP production. Figure created with www.BioRender.com

Rotenone is rapidly absorbed across the gill epithelium and blocks oxygen use by the cells. Mitochondria is the main target of rotenone (Fig. 1) (Chen et al. 2003) as it inhibits the transfer of electrons from complex I to ubiquinone. The subsequent leakage of electrons from complex I leads to partial reduction of oxygen to form superoxide ion (^·^O_2_^−^), the precursor of most other reactive oxygen species (ROS). Subsequently, ^·^O_2_^−^ is quickly dismutated to hydrogen peroxide (H_2_O_2_) by superoxide dismutase 1 (SOD) in the mitochondrial matrix. H_2_O_2_ is then converted to water by catalase and/or glutathione (GSH) peroxidase. Both ^·^O_2_^−^ and H_2_O_2_ are considered as harmful to the cell and may induce oxidative DNA damage, ER stress and apoptosis.

ROS is frequently formed following exposure to environmental stress factors like toxic compounds and infections agents. Species differences in responses to ROS may therefore result in differences in tolerance to ROS-inducing factors. In accordance with this assumption, it was shown that exposure to rotenone affected the gene expression related to oxidative stress differently in the sensitive species silver carp (*Hypophthalmichthys molitrix)* compared to the much more tolerant species bigmouth buffalo (*Ictiobus cyprinellus)* (Amberg et al. 2012). The authors concluded that the different mechanisms these fish species use to handle oxidative stress are important for the species differences in sensitivity to rotenone. It is therefore essential to know how your model system responds to external stimuli to make relevant interpretations of the results.

In this study, we assessed ROS handling of two salmonid gill epithelial cell lines, the well-known RTgill-W1 (Bols et al. 1994; Fischer et al. 2019) cell line from Rainbow trout *(Oncorhynchus mykiss)* and the newly established ASG-10 (Gjessing et al. 2018) from Atlantic salmon exposed to rotenone.

## Materials and methods

### Cell culture and treatments

RTgill-W1 cell line was obtained from American Type Culture Collection (ATCC) and grown in Leibowitz’s L-15 Glutamax (Gibco, Thermo Fisher, Waltman, MA, USA) supplemented with 10% FBS (EU standard, Biosera, Nuaille, France) and 1% penicillin/streptomycin (Lonza, Basel, Switzerland). The cells were cultured at 19 °C in a non-ventilated cell culture flask and sub-cultured 1:2 once every 10th day using trypLE (Gibco). For experiments, the cells were seeded (132,000/cm^2^) 2 days before the experiments. The cell number gave full confluence at the day of experiment. The ASG-10 cell line was previously made by our lab (Gjessing et al. 2018) and grown in Leibowitz’s L-15 Glutamax medium supplemented with 10% FBS, 1% penicillin/streptomycin and 30 µM β-merceptaethanol (Sigma-Aldrich, St-Louis, MO, USA). The cells were cultured at 19 °C in a non-ventilated cell culture flask and sub-cultured 1:2 every 10^th^ day using trypLE. For experiments, the cells were plated out as described for RTgill-W1, using cell culture medium without β-merceptaethanol. Rotenone (Sigma-Aldrich) was dissolved in DMSO and stock solutions (0.05, 0.5 and 5 mM) stored at − 20 °C. Unpon exposure, the medium was replaced with exposure medium (L-15 with 10% FBS and 1% penicillin/streptomycin) with or without rotenone and inhibitors as indicated. The final concentration of DMSO in the cell culture was 0.1%. Appropriate controls containing the same amount of solvent were included in each experiment. *N*-actyl-l-cysteine (NAC; Sigma-Aldrich) and l-Buthionine-S,R-sulfoximine (BSO; Bio-techne, Minneapolis, MI, USA) were prepared fresh in front of each experiment and dissolved in exposure medium as described above. The NAC solution was pH adjusted to 7.4.

### Metabolic activity, viability

Metabolic activity/ viability of the cells was measured using the Alamar Blue assay according to the manufacturer’s protocol (Invitrogen, Thermo Fisher). The dark blue oxidized form of Alamar Blue (resazurin) is reduced to a highly fluorescent form (resorufin) by mitochondrial or cytoplasmatic enzymes (Rampersad 2012). The measured fluorescence intensity is thus proportional to the number of viable cells. The fluorescence; Excitation (Ex) 555 nm/Emission (Em) 585 nm was quantified using Spectramax i3x plate reader (Molecular Devices, San Jose, CA, USA).

### Cytotoxicity

CellTox green is a non-toxic dye that stains DNA of cells with impaired membrane integrity. The binding with DNA produces a fluorescence signal that is proportional with cytotoxicity (necrotic and late apoptotic cells). CellToxTM Green Dye (1:2000; Promega, Madison, WI, USA) was added to the cells as described by the manufacturer and fluorescence (Ex 485 nm/Em 520 nm) quantified by Spectramax i3x plate reader. To ensure a representative readout of the fluorescent adherent cells, 37 different points were read in each well using the well scan function of the plate reader.

### MitoTracker Red staining

The cells were seeded on microscopy polymer coverslips (Ibidi, Gräfelfing, Germany) and treated with rotenone (0.5 µM, 2 h). The cells were stained with MitoTracker Red CMXRos (Molecular Probes, Invitrogen; 40 nM) in L-15 culture medium for 20 min at 19 °C. The staining solution was replaced with phenol red free L-15 medium with 10% FBS and 1% penicillin/streptomycin and mitochondria visualized by confocal microscopy (Zeiss LSM710, 63 × NA 1.4 oil objective).

### Mitochondrial membrane potential

Mitochondrial membrane potential was determined using 5,5′,6,6′-tetrachloro-1,1′,3,3′-tetraethylbenzimi-dazolylcarbocyanine iodide (JC-1; Biotium, Fremont, CA, USA), which accumulates and aggregates in intact mitochondria, emitting bright red fluorescence (Ex 497 nm/Em 595 nm). With disruption of the mitochondrial membrane potential, these aggregates do not form, and JC-1 remains in its monomeric form emitting a green fluorescence (Ex497/Em528). *Microscopic analysis:* The cells were seeded on microscopy polymer coverslips (Ibidi) and treated with rotenone (0.5 µM, 2 h). The cells were stained with JC-1 according to the protocol for 20 min at 19 °C. The staining solution was removed then and phenol red free L-15 medium were added before visualized by confocal microscopy (Zeiss LSM710, 63 × NA 1.4 oil objective, using Ex 488 nm/Em 493–582 nm (green) and Ex 488 nm/Em 599–758 nm (red). *Flow cytometric analysis:* Rotenone-treated cells, for some unknown reason, did not detach from the plastic well by using trypLE (or trypsin). To evaluate the mitochondrial membrane potential by flow cytometry, the cells were therefore detached by using trypLE as described in 2.1, re-suspended the cells in complete cell culture medium (1 × 10^6^/ml) and treated with rotenone (0.5 µM, 2 h) in an eppendorph tube. After exposure, the cells were washed once in PBS and stained with JC-1 and propidium iodide (5 µg/ml, Thermo Fisher) according to the protocol in serum free L-15 cell culture medium for 20 min at 19 °C. A minimum of 20 000 cells were then analysed by flow cytometry (Accuri C6, BD, Franklin Lakes, NJ, USA). The ratio of red fluorescence (Ex 488 nm/Em 564–606 nm) and green fluorescence (Ex 488 nm/Em 515–545 nm) were used to determine healthy versus depolarized mitochondria. Necrotic cells (PI positive cells; Ex 488 nm/Em 670 nm LP) were excluded from the analysis by gating.

### ROS production

ROS production was detected by using the oxidation-sensitive fluorescent probe, CM-H_2_DFDA (Molecular Probe, Invitrogen). The cells were first pre-incubated with CM-H_2_DFDA (2 µM) in PBS for 30 min, followed by exposure to rotenone in complete cell culture medium for 2 h. The medium was then replaced with HBSS and fluorescence (Ex 485 nm/Em 520 nm) analysed by Spectramax i3x plate reader. To ensure a representative readout of the fluorescent adherent cells, 37 different points were read in each well using the well scan function of the plate reader. Equal cell number in each well were verified by staining the nuclei with DRAQ5 (nuclear staining, 1:500; Thermo Fisher) for 30 min at room temperature, and cell number were counted by the spectramax i3x plate reader equipped with a microscopic module (MiniMax300Imaging Cytometer, Molecular Devices). For flow cytometric analyses, the cells were treated (CM-H_2_DFDA pre-incubation and rotenone exposure) in eppendorph tubes for reasons as described in 2.5. When using the efflux-pump inhibitor probenicid (1 mM), it was used during the CM-H_2_DFDA staining and rotenone exposure.

### Cellular glutathione

The relative concentrations of intracellular glutathione were determined by using using monobromobimane (mBBr), which binds to the SH group of the reduced form of glutathione (GSH), thereby forming a fluorescent adduct (Cotgreave and Moldeus 1986). After exposure, the cells were incubated with 40 µM mBrB (Sigma-Aldrich) in diluted in PBS with 2% FBS for 20 min at room temperature, and analysed by Spectramax i3x plate reader. To ensure a representative readout of the fluorescent adherent cells, 37 different points were read in each well by using the well scan function of the plate reader. For flow cytometry, the cells were trypsinated, resuspended in PBS and incubated with mBrB (20 µM) for 15 min. At least 10 000 cells were analysed by flow cytometry (Ex 405 nm/Em 515–545 nm) using NovoCyte Flow cytometer (Aglient, Santa Clara, CA, USA).

### Catalase activity

Catalase activity was measured by using Catalase Colorimetic Activity Kit (EIACATC Invitrogen, Thermo Fisher) as described by the manufacturer. Briefly, cells were seeded in 6 cm dishes as described above. The next day the cells were exposed for rotenone for 2 h, washed 2× with cold PBS and scraped in 250 µl cold Assay Buffer. The cell lysate was then frozen down (− 80 °C) until the next day. The cell lysate was then homogenizated by using a QIAshredder (Qiagen, Hilden, Germany), centrifuged (10,000×*g*, 15 min) and the supernatant collected. The assay was then further performed as described in the kit. Protein concentration of the catalase lysates were measured by using DC Protein Assay (Bio-Rad, Hercules, CA, USA).

### Statistical analysis

The data analyses were performed using GraphPad Prism version 9.0.1(151). Statistical significance (p < 0.05) was assessed using ANOVA, followed by Holm–Sidak post-test. Standard error of the mean (SEM) is used as the mean of several independent experiments with three or more replicates are shown.

## Results

### Metabolic activity and cytotoxicity

**Fig. 2:**
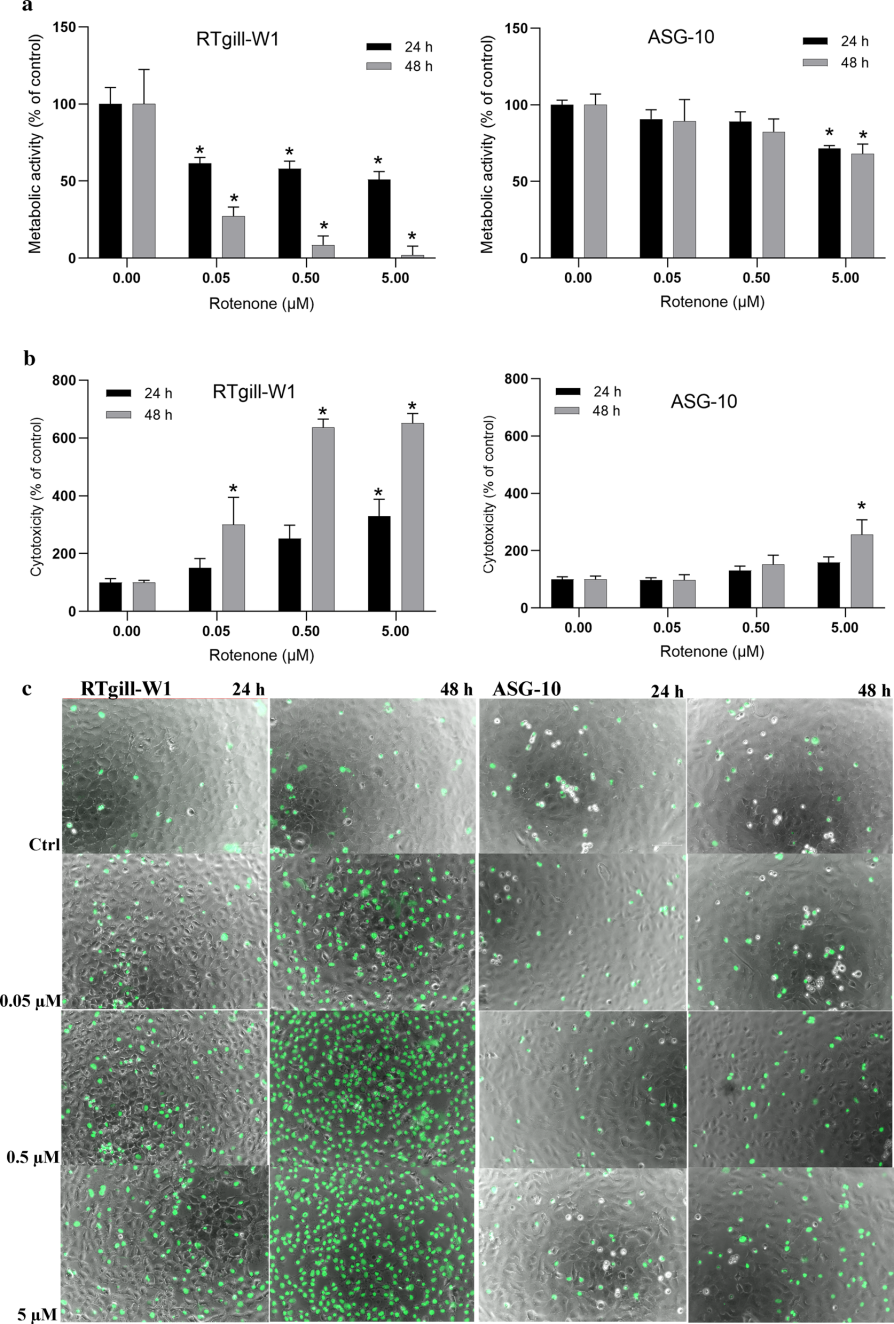
Rotenone reduced viability. The cells were treated with rotenone (0.05–5 µM) for 24 or 48 h and analysed for metabolic activity by Alamar Blue (**a**) and cytotoxicity by Celltox Green (**b**). The graphs represent data from 3–4 independent experiments, expressed as mean ± SEM. Significance difference (p < 0.05) compared to control is indicated with an asterix (*). **c** Celltox green stained cells visualized by fluorescence microscopy. Green = necrotic / late apoptotic cell. (Color figure online)

We compared rotenone-induced toxicity in the two salmonid gill epithelial cell lines, RTgill-W1 (Rainbow trout) and ASG-10 (Atlantic salmon). The cells were treated with rotenone (0.05–5 µM) for 24 or 48 h and the metabolic activity measured by the Alamar Blue assay (Fig. 2a). Rotenone reduced the metabolic activity by 50% already at 0.05 µM after 24 h in the RTgill-W1 cells. In contrast to this, a statistically significant reduction was first observed at 5 µM rotenone exposure after 24 h exposure in the ASG-10 cells. Furthermore, in contrast to the RTgill-W1 cells, no measurable difference between 24 and 48 h exposure was seen in the ASG-10. Next, we investigated rotenone induced cytotoxicity. Here we used the Celltox Green assay staining DNA in cells with compromised plasma membrane integrity. After 24 h exposure, rotenone had moderate cytotoxic effect on both cell lines, with RTgill-W1 being more sensitive than ASG-10 (Fig. 2b). At this time point, the Alamar Blue assay is more sensitive than the Celltox Green assay, indicating that the metabolic activity machinery is a primary target of rotenone and not the plasma membrane. After 48 h, rotenone was more cytotoxic to the RT-gill-W1 cells than to the ASG-10 cells at all tested concentrations. Surprisingly, ASG-10 were not very sensitive to rotenone even at 5 µM. However, following even longer exposure of rotenone (5 µM, 96 h), more cell death was observed also in the ASG-10 cells (data not shown).

**Fig. 3:**
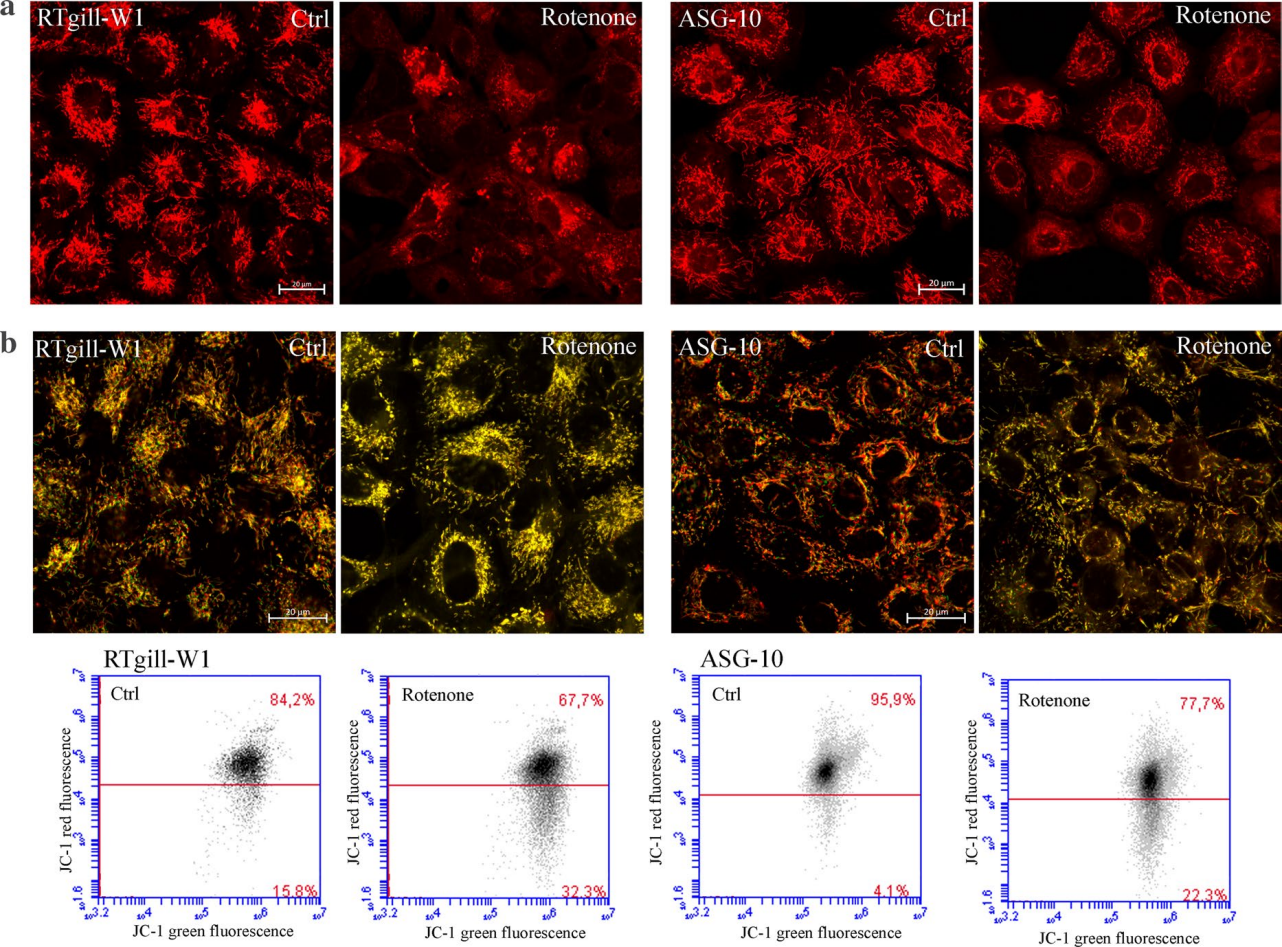
Rotenone induce mitochondrial changes. The cells were treated with Rotenone (0.5 µM for 2 h), stained with MitoTracker Red (**a**) or JC-1 (**b**) and visualized by confocal microscopy (63×, scale bar = 20 µm) or analysed by flow cytometry. (Color figure online)

### Mitochondrial morphology, membrane potential and ROS

Since the primary target of rotenone is the mitochondria, our next step was to investigate the morphology of the mitochondria and their response to rotenone. The MitoTracker Red is a fluorescent dye that accumulates in normal mitochondria with a highly negative mitochondrial membrane. Interestingly, in untreated RTgill-W1 cells, the mitochondria appear more fragmented compared to the mitochondria of ASG-10 cells, which are longer and more spaghetti-like (Fig. 3a). Rotenone exposure (0.5 µM, 2 h) altered the mitochondria morphology in both cell lines, but they appear to be even more fragmented in the RTgill-W1 cells compared to ASG-10. To investigate the impact of rotenone on the mitochondria membrane potential (∆Ψm) we used the specific JC-1 probe. In normal mitochondria with high ∆Ψm, JC-1 forms aggregates that emit red light. In cells with disrupted, ant low ∆Ψm, the JC-1 emit green light. Microscopic and flow cytometry analysis (Fig. 3b) confirmed reduced ∆Ψm after rotenone treatment in both cell lines, with RTgill-W1 seemingly somewhat more sensitive than the ASG-10 cells.

**Fig. 4:**
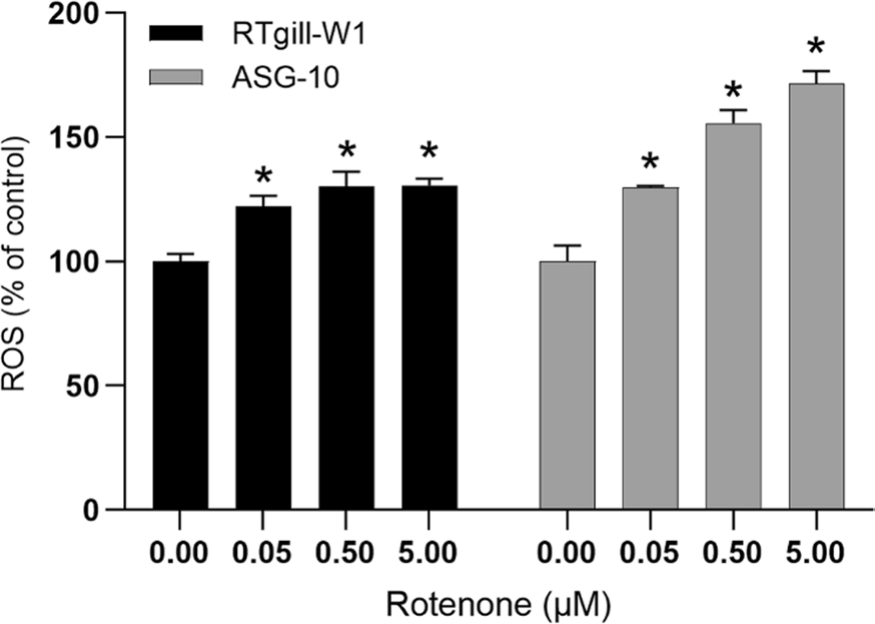
Rotenone induced ROS. The cells were treated with Rotenone 0.5–5 µM for 2 h and analysed for ROS production by using the CM-H_2_DFDA probe. Data are expressed as mean ± SEM of 4 independent experiments. Significance difference (p < 0.05) compared to control is indicated with an asterix (*)

Rotenone is known to produce ROS due to its interference with the mitochondrial electron transport chain. To examine the ROS production in RTgill-W1 and ASG-10, we used the CM-H_2_DFDA probe, which is a commonly used indicator of ROS. As expected, rotenone induced ROS production in both cell lines (Fig. 4). Interestingly, opposite of the outcome of metabolic activity and cytotoxicity measurements, rotenone induce a larger response on ROS production in ASG-10 cells than in the RTgill-W1 cells. However, it is known that efflux pumps are able to pump the staining probe out of the cells, leading to lower staining intensity (Sieprath et al. 2017). To investigate if this was the case in RTgill-W1 cells, we used the efflux pump inhibitor probenicid (1 mM, Biotium) together with the staining solution. Probenecid considerably increased the apparent ROS formation on the RTgill-W1 cells (Supplementary). Thus, we could not quantitatively compare the production of ROS in the two cell lines, we could only conclude that both cell lines produce ROS in response to rotenone.

### Antioxidant defence

**Fig. 5:**
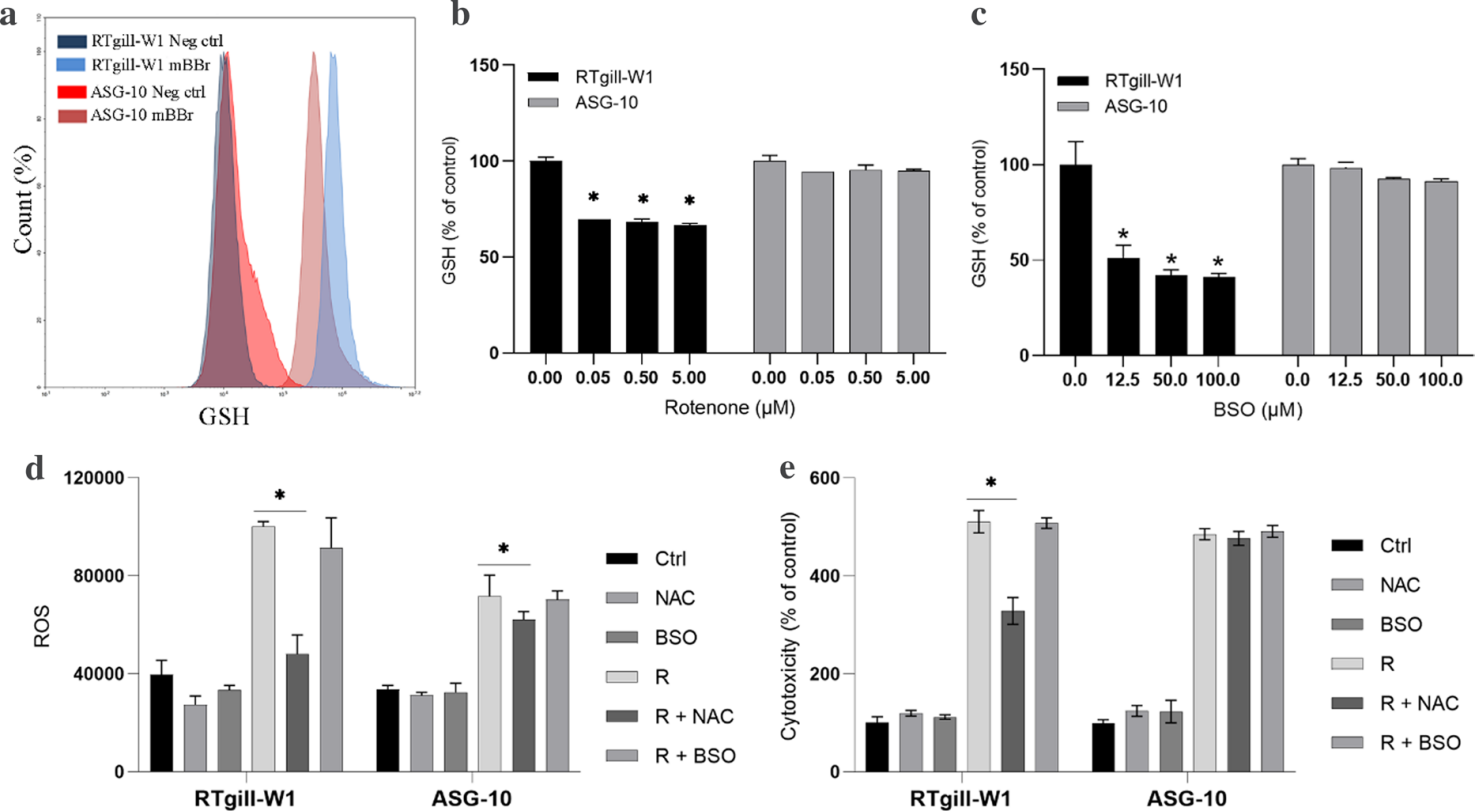
Intracellular GSH. **a** Cells, unstained (Neg ctrl) or stained with mBBr, a marker for GSH, were analysed by flow cytometry. **b** The cells were treated with rotenone (0.05–5 µM) for 2 h and analysed for GSH by using the mBBr probe and the plate reader. **c** The cells were treated with BSO (12.5–100 µM) for 24 h and analysed for GSH (mBrB) by plate reader. The cells were pre-treated with BSO (50 µM) or NAC (10 mM) for 24 h, exposed to rotenone (R), 0.05 µM for the RTgill-W1 cells and 5 µM for the ASG-10 cells, and analysed for ROS after 2 h (**d**) or exposed for 24 h and analysed for cytotoxicity by using the Celltox green assay (**e**). The graphs (**b, c, e**) represent mean ± SEM of 3 independent experiments. The graph **d** represent mean ± SD of 3 parallel incubations. Significance difference (p < 0.05) compared to control is indicated with an asterisk (*). (Color figure online)

GSH is usually highly abundant in cells and is needed to maintain the cellular oxidative balance by reducing H_2_O_2_ to H_2_O and glutathione disulphide (GSSG). Therefore, we tested the hypothesis that differences in antioxidant defence was the cause the differences in cytotoxicity. The level of GSH in the cells were investigated by using monobromobimane (mBBr), which binds to the SH group of GSH (reduced form) and forms a fluorescent adduct (Cotgreave and Moldeus 1986). The amounts of GSH-adducts in single cells were then analysed by flow cytometry. The RTgill-W1 cells contained somewhat more GSH than the ASG-10 cells (Fig. 5a). Rotenone treatment led to a great GSH depletion in the RTgill-W1 cells while no such effect could be observed in the ASG-10 cells (Fig. 5b). This implies that the RTgill-W1 cells deplete its GSH-pool faster than the ASG-10 cells and subsequently are more vulnerable to the cytotoxic effects of rotenone induced ROS. This also corresponds with the toxicity data, with RTgill-1W cells being more sensitive to rotenone-induced cell death than ASG-10 cells. To investigate the role of GSH in antioxidant defence in more detail, we used l-Buthionine-S,R-sulfoximine (BSO), which is a GSH depleting agent that inhibits γ-glutamylcysteine synthetase, an essential enzyme in GSH synthesis. Here, BSO reduced the GSH level in the RTgill-W1, while only a very small, non-significant reduction of GSH was seen in the ASG-10 cells (Fig. 5c). Furthermore, BSO alone did not induce any cytotoxicity in either of the gill cells. Also, pre-incubation with BSO before rotenone exposure did not enhance the cytotoxicity of rotenone (Fig. 5e). This implies that BSO together with rotenone did not decrease the GSH level further and thus did not increase the cytotoxicity of rotenone in the RTgill-W1 cells. In contrast, *N*-acetyl-l-cysteine (NAC), a precursor of l-cysteine that results in GSH elevation, and thus often used as an antioxidant, significantly reduced rotenone induced cytotoxicity in the RTgill-W1 cells but had no effect in the ASG-10 cells (Fig. 5e). Similarly, NAC reduced the rotenone-induced ROS in RTgill-W1 cells, but had only minor effect on the ASG-10 cells. BSO had no effects on either cell lines (Fig. 5d). Taken together, this indicates that GSH depletion together with ROS production is an important mediator of rotenone induced cytotoxicity.

**Fig. 6:**
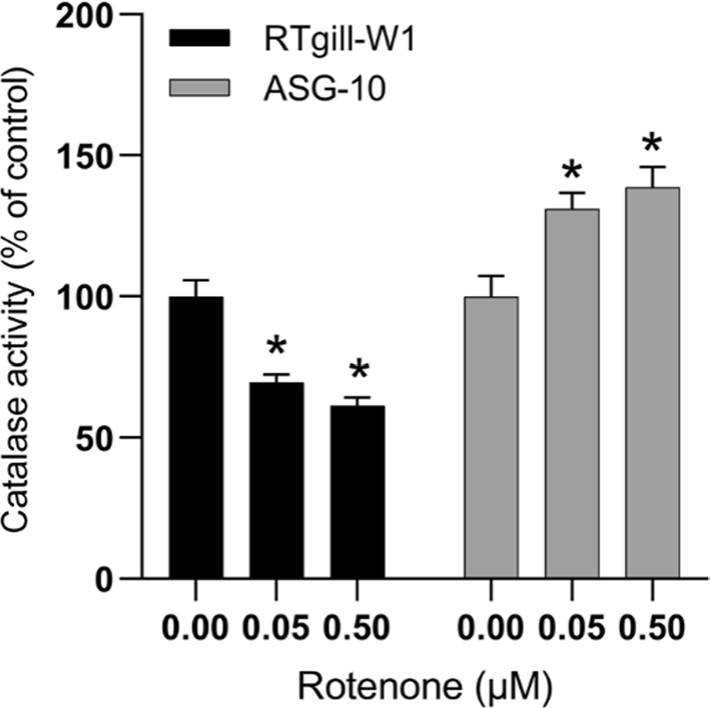
Effects of rotenone exposure on catalase activity. The cells were treated with rotenone for 2 h, lysed and measured for enzyme activity. Data are expressed as mean ± SEM of 3–4 independent experiments. Significance difference (p < 0.05) compare to rotenone exposure is indicated with an asterisk (*). The data is normalized to protein level in addition to control

To investigate this further, we measured the activity of the H_2_O_2_ scavenger catalase. Following rotenone exposure (0.05 and 0.5 µM, 2 h) the catalase activity in RTgill-W1 was lower after rotenone exposure, while the opposite was observed in the ASG-10 cells (Fig. 6).

## Discussion

ROS can be generated upon infections as well as following toxicological challenges. Knowledge of how RTgill-W1 and ASG-10 respond to ROS-inducing stressors is therefore essential to make relevant interpretations when assessing ROS-inducing triggers. Here we report that rotenone, a known inducer of ROS, induces highly different responses in the salmonid gill cell lines RTgill-W1 (Rainbow trout) and ASG-10 (Atlantic salmon). Rotenone affected mitochondria and triggered production of ROS in both cell lines. However, rotenone reduced metabolic activity and induced cell death to a much higher degree in RTgill-W1 than in ASG-10. GSH and catalase were depleted in RTgill-W1, while in ASG-10, no changes was seen in GSH levels, while the catalase activity was increased. These findings may partly explain the observed difference in sensitivity between RTgill-W1 and ASG-10 to rotenone.

The RTgill-W1 cells are significantly more sensitive towards rotenone than ASG-10, measured with both metabolic activity assay (Alamar blue) and cytotoxicity assay (Celltox green). After 24 h exposure, more pronounced effects were observed in metabolic activity assay than for the cytotoxicity assay. This indicates that rotenone is either affecting metabolic activity in the cells or cell proliferation rather that causing cell death. This is not surprising since rotenone is known to interfere with the electron transport chain and hence cellular energy production. In our study rotenone reduced the viability of RTgill-W1 by 40% at 0.05 µM, while the ASG-10 cells reduced the viability by 28% at 5 µM. Several in vitro studies have shown reduced viability after rotenone exposure with variable sensitivity. In HepG2 cells morphological changes could be observed after 24 h at 25 µM, while the IC_25_ (MTT assay; equivalent to the Alamar blue assay), was about 50 µM (Siddiqui et al. 2013). In contrast, 10 µM rotenone reduced the viability (MTT assay) by 37% in the neural cell line NSC34 after 24 h (Jung et al. 2015). Also, in human neuroblastoma SH-SY5Y dopaminergic cells 5 µM rotenone reduced the viability (MTT assay) by 60% after 24 h exposure (Ma et al. 2018). Compared to these studies the RTgill-W1 is indeed highly sensitive to rotenone while the sensitivity of the ASG-10 cell line is in the range reported from other cell lines.

The differences in sensitivity towards rotenone might be explained by differences in the mitochondria, the primary target of rotenone. Microscopic evaluation indicated that the mitochondria in unexposed RTgill-W1 cells are slightly more fragmented compared to mitochondria in the ASG-10 cells. Mitochondria are dynamic organelles that constantly undergo fusion and fission. The difference in mitochondrial morphology between the two cell lines might therefore indicate different balance between these two processes as rotenone is a well-known inducer of ROS and mitochondrial fragmentation (Frank et al. 2012; Passmore et al. 2017). While mitochondrial fusion tends to increase the bioenergetics efficiency and maintaining the ATP production, mitochondrial fragmentation is associated with decreased fusion and increased fission and characterized by a large number of smaller mitochondria. Furthermore mitochondrial fragmentation is linked to increased oxidative stress, mitochondrial depolarization and reduced ATP production (Liu et al. 2020).

Mitophagy is a specific form of autophagy that eliminate damaged mitochondria in cells (Frank et al. 2012; Liu et al. 2020). The interplay with mitochondrial dynamics ensuring functional mitochondria. Rotenone has also been shown to trigger mitophagy, induced by mild oxidative stress in a mitochondrial fission-dependent manner. Interestingly, it has been suggested that mitophagy functions as a negative regulatory feedback mechanism by reducing mitochondrial derived ROS production (Kurihara et al. 2012). Reduction of the ∆Ψm is a common effect of rotenone exposure (Moon et al. 2005) and has been associated with mitophagy. Mitochondria resulting from a fission event that are unable to re-establish ∆Ψm (an indication of damage), fails to reintegrate to the fusing pool and is likely an early event preceding mitophagy (Twig et al. 2008). Furthermore, even though reduced ∆Ψm is an apoptotic signal, it has been reported to be a fully reversible event and does not irreversibly commit cells to die (Minamikawa et al. 1999; Sunaga et al. 2014). Our findings suggests that the RTgill-W1 cells has somewhat more fragmented mitochondria than the ASG-10 cells, and are more prone to depolarization in response to rotenone. A hypothesis could therefore be that the ASG-10 cells have higher amount of mitophagy and mitochondrial recovery than the RTgill-W1 cells and thus less sensitive to the rotenone-induced toxicity. However, this remain to be investigated. Furthermore, based on the very high sensitivity of the RTgill-W1 cell line in combination with the morphological appearance of the mitochondria we may speculate that these cells may be a less suitable model for stressors affecting this organelle, but comparisons with the mitochondria in healthy primary gill cells would be desirable.

In a previous study it was reported that the microsomal uncopling protein 2 (UP2) was induced 85 fold in the rotenone-resistant species bighead carp, but not in the more sensitive silver carp (Amberg et al. 2012). UCP2 is a member of inner mitochondrial membrane proteins that dissipates the mitochondrial proton gradient by transporting H^+^ across the inner membrane, thereby generating heat, stabilizing the inner mitochondrial membrane potential and reducing the formation of ROS (Pierelli et al. 2017). Interestingly, a downregulation of UCP2 is associated with increased mitochondrial fission (He et al. 2020). These findings suggest a link between rotenone induced fragmentation (fission), increased mitophagy, reduced ∆Ψm and expression of UCP2.

As expected, rotenone increased the production of ROS in both cell lines, but probably due to different expression of efflux pumps and therefore uncertainties in the assay (Sieprath et al. 2017) any potential differences could not be quantified. Cells have a complex antioxidant defence network to limit any damaging effects of ROS. GSH is one key factor in the antioxidant defence system. GSH protects the cells from ROS by reducing the ROS forms and is converted to its oxidised form GSSH. Depletion of cellular antioxidant defences, such as GSH, results in accumulation of ROS, loss of mitochondria function and thus induction of cell death. Rotenone-induced GSH depletion is observed in several studies and is considered to play a crucial role in the induction of cell death (Sanchez-Reus et al. 2005; Siddiqui et al. 2013). In the RTgill-W1 cells, treatment with rotenone led to GSH depletion, while addition of the GSH precursor NAC, significantly reduced rotenone induced ROS production and dell death. These observations indicates that GSH plays an important role in the handling of ROS as well as cytotoxicity in the RTgill-W1 cell line. Interestingly, BSO induced depletion of GSH had no effect on rotenone induced ROS nor cell death, which indicates that it was not possible to decrease the GSH level further and thus no increase in cytotoxicity compare to rotenone alone in the RTgill-W1 cells. In contrast to this, the ASG-10 cells did not alter the GSH levels in response to rotenone. In accordance to this, the addition of NAC had very low or no effect on ROS production and cell death. Also, addition of BSO only very slightly reduced the GSH level in the ASG-10 cells, and no effects were observed at the levels of ROS and cell death. Based on our results it seems that GSH depletion and ROS production is important factors in rotenone induced cytotoxicity in the two fish gill cell lines, and the differences reflect their different sensitivity towards rotenone induced cell death.

Catalase is another important enzyme to reduce intracellular ROS levels. It dissociates hydrogen peroxide into molecular oxygen and water. Similar to GSH, reduced catalase activity is considered to be important in rotenone-induced cell death (Siddiqui et al. 2013). We found that rotenone induced depletion of catalase activity in the RTgill-W1 cell line, while it increased the catalase activity in the ASG-10 cell line. These findings are in line with the cytotoxicity data. Higher cytotoxicity in the RTgill-W1 cells imply that the protective mechanisms are reduced, while in the more robust ASG-10 cell line the catalase activity still is involved in the elimination of hydrogen peroxide after rotenone exposure.

Overall, the two gill cell lines from the closely related salmonids appear to have different tolerance levels to ROS and to differ in ROS response. These differences need to be taken into consideration when choosing cell lines for studies of ROS-inducing processes and for the interpretation of the results. A comparison of the ROS handling abilities in the cell lines with gill cells from live fish would reduce uncertainties related to the relevance of the models for in vivo effects of cellular processes involving ROS-formation and handling processes.

## Supplementary Information

Below is the link to the electronic supplementary material.

Supplementary file1 (TIF 42552 KB)
