# Supplementary figures and images for: The gill epithelial cell lines RTgill-W1, from Rainbow trout and ASG-10, from Atlantic salmon, exert different toxicity profiles towards rotenone
Source: Cytotechnology. 2022 Nov 17;75(1):63–75. doi: 10.1007/s10616-022-00560-0 (PMC9880101; doi:10.1007/s10616-022-00560-0)

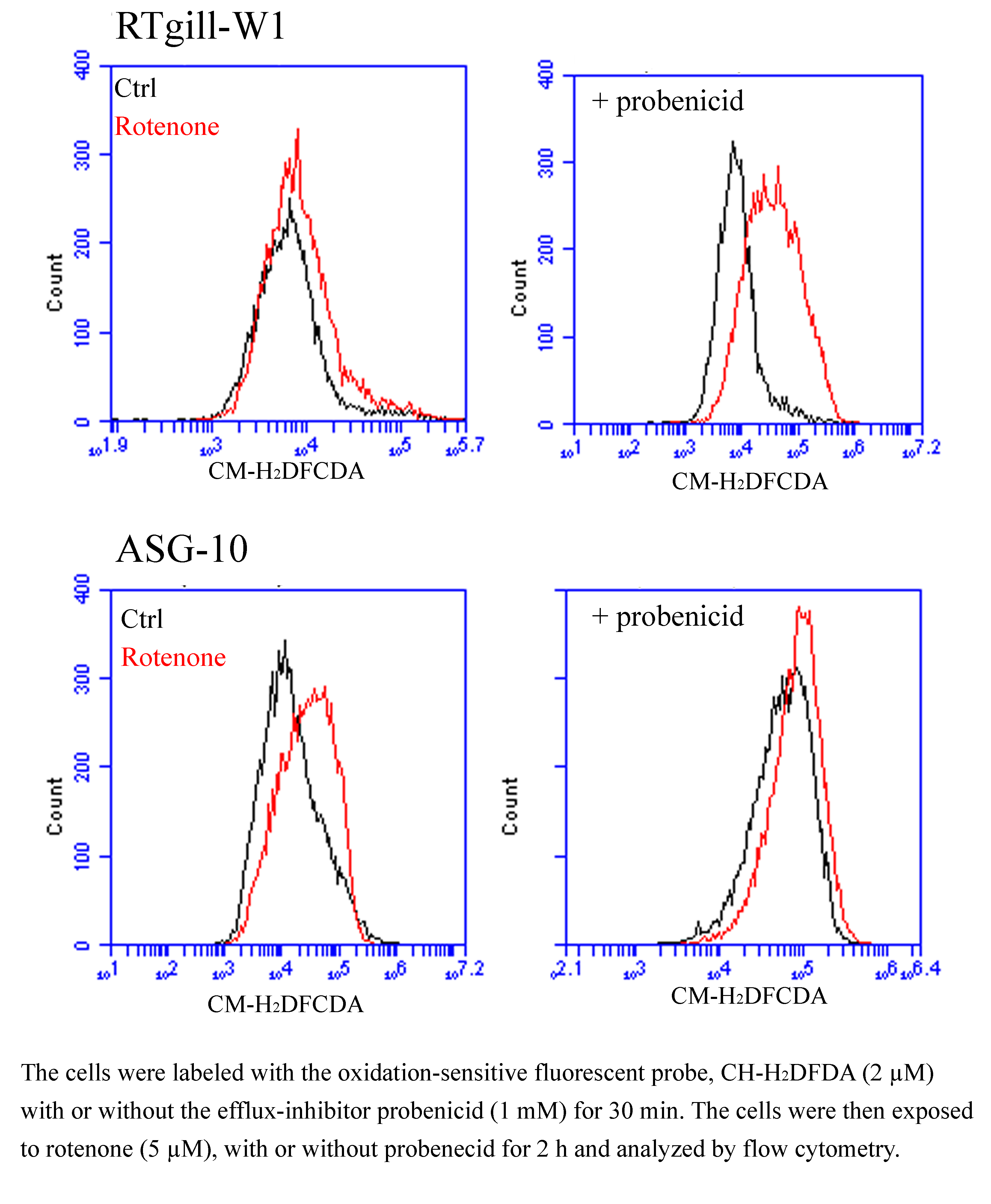

Supplement: Supplementary file 1 — Supplementary file1 (TIF 42552 KB) [file 10616_2022_560_MOESM1_ESM.tif]
